# Synchronous Mediastinal and Central Nervous System Involvement in Rosai-Dorfman Disease: A Case Report

**DOI:** 10.7759/cureus.39984

**Published:** 2023-06-05

**Authors:** Carolina Soto-Davila, Rachel Klapper, Jacob Afude, Guillermo Sangster, Carlos Previgliano

**Affiliations:** 1 Radiology, Louisiana State University Health Sciences Center, Shreveport, USA; 2 Pathology, Louisiana State University Health Sciences Center, Shreveport, USA

**Keywords:** rosai-dorfman disease, mediastinum, brain, pet-ct, mri, histiocytosis

## Abstract

Rosai-Dorfman disease (RDD) is a rare disorder characterized by the proliferation and accumulation of histiocytes, primarily within lymph node sinuses. Uncommonly, other extranodal sites, such as the central nervous system, can also be affected. Here, we document the case of a 61-year-old woman presenting with dizziness, confusion, and headaches. Magnetic resonance imaging (MRI) showed an extra-axial avidly enhancing mass in the left parietal region presumed to be a meningioma based solely on its imaging appearance. The patient underwent surgical resection, and histopathological examination showed enlarged histiocytes positive for S100, CD68, and CD163 and negative for CD1a, consistent with RDD. She was followed up with a positron emission tomography/computed tomography (PET/CT) to evaluate other disease activity sites. A single mediastinal node was identified adjacent to the atriocaval junction intensely fluorodeoxyglucose avid. The patient underwent robotic node excision, with pathology analysis compatible with RDD. We emphasize the need to increase recognition of RDD on differential brain lesions, especially meningiomas, and suggest PET/CT as a valid tool to search for other disease activity lesions.

## Introduction

Rosai-Dorfman disease (RDD), also known as sinus histiocytosis with massive lymphadenopathy [1], is a rare non-Langerhans cell histiocytic disorder with an unknown etiology [2] characterized by the accumulation of activated histiocytes within affected tissues [3]. It was initially described by Destombes in 1965 as “adenitis with lipid excess” [4]. In 1969, pathologists Juan Rosai and Ronald Dorfman recognized the disease as a histiolymphoproliferative disease of the lymph nodes [1]. The disease is more frequently seen in children and young adults, especially males [5]. The underlying causes of RDD remain unclear. Cervical lymphadenopathy is the most common presentation of RDD [6], with extranodal compromise reported in less than 50% of the cases. Therefore, histopathologic evaluation is crucial for diagnosis. In many cases, RDD is considered a benign disease with spontaneous regression. However, some patients may encounter an aggressive course and need systemic treatments such as steroids, chemotherapy, and radiotherapy [7].

## Case presentation

**Figure 1:**
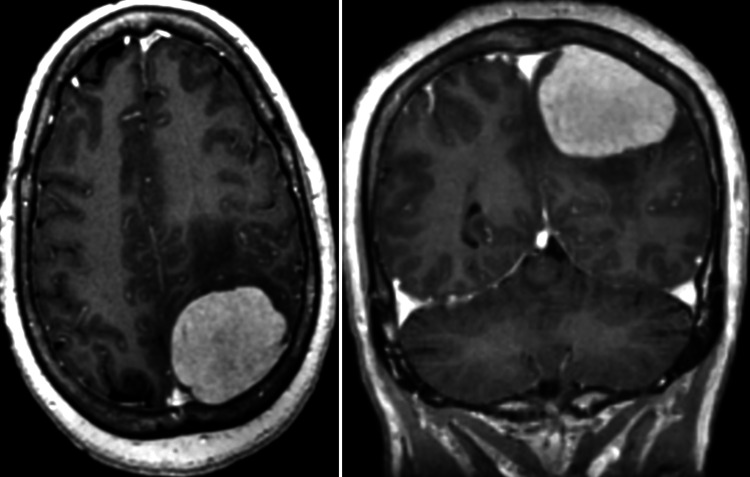
Magnetic resonance imaging (MRI) of the brain with contrast. MRI of the brain with contrast shows a homogeneously enhancing and well-defined dural-based mass at the left parietal convexity, causing mass effect upon the underlying brain parenchyma. The lesion measures 5 × 4 × 3.3 cm and is associated with mild hyperostosis of the overlying parietal bone. The mass demonstrated a low signal on the non-contrast T1-weighted images and intermediate signal intensity on T2-weighted images (not shown). There is also left atrial effacement and minimal vasogenic edema.

**Figure 2:**
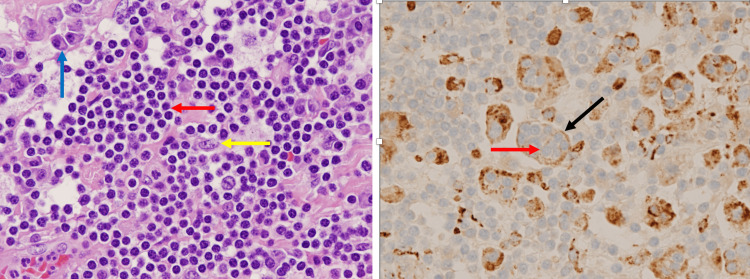
Pathology. (Left panel) Plasma cells (blue arrow). Histiocytes (yellow arrow). Lymphocytes within the cytoplasm of histiocytes (red arrow) which is called emperipolesis (hematoxylin and eosin ×200). (Right panel) Histiocytes are highlighted by membranous CD68 staining (black arrow). Engulfed lymphocytes in the cytoplasm of histiocytes (red arrow) (CD 68 immunostain ×200).

A 61-year-old female with no significant medical history presented with dizziness, confusion, headaches, and pain over the left side of her body for the past year. During the hospital visit, magnetic resonance imaging (MRI) of the brain showed a dural-based, avidly enhancing mass at the left parietal convexity measuring 5 × 4 × 3.3 cm, most likely compatible with meningioma (Figure 1). The patient underwent left-sided craniotomy with resection of the mass, and histopathologic examination showed abundant histiocytes, plasma cells, and small lymphocytes. The histiocytes were enlarged with a vesicular nuclear membrane and prominent nucleoli. In addition, there were occasional Russel bodies and intracytoplasmic lymphocytes engulfed by enlarged histiocytes. Immunostaining showed histiocytes positive for S100, CD68, and CD163 and negative for CD1a (Figure 2). The morphology and immunostaining pattern was consistent with RDD.

**Figure 3:**
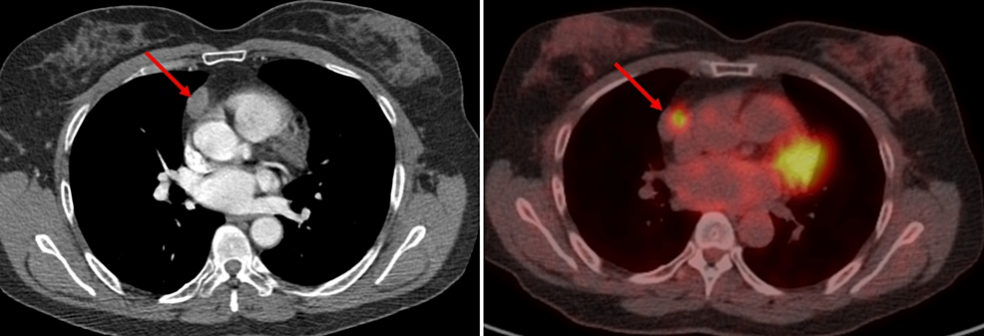
Fluorodeoxyglucose (FDG) positron emission tomography/computed tomography (PET/CT) axial images. FDG PET/CT axial images of the chest show a 2.3 × 1.4 cm right mediastinal node adjacent to the atriocaval junction which is intensely FDG-avid with a maximum standardized uptake value of 7.4.

After the meningeal pathology findings, the patient underwent a positron emission tomography/computed tomography (PET/CT) to evaluate other sites of disease activity, revealing a solitary intensely fluorodeoxyglucose (FDG)-avid node in the right-sided mediastinum adjacent to the atriocaval junction (Figure 3). The patient received robotic excision of the mediastinal node, and histopathology evaluation was also consistent with RDD. After the procedure, the patient remained asymptomatic and without nodal recurrence.

## Discussion

RDD is an uncommon disease with a prevalence of 1:200,000 and an estimated 100 new cases annually in the United States [8]. RDD can occur sporadically or be associated with familial syndromes such as H syndrome and autoimmune lymphoproliferative syndrome. It also has been associated with malignant (leukemia, lymphoma, cutaneous clear-cell sarcoma) and autoimmune disorders (systemic lupus erythematosus, idiopathic juvenile arthritis, autoimmune hemolytic anemia) [3].

The etiology of RDD is still unknown. Some theories considered an immune dysfunction (abnormal response to an undefined antigen or an alteration in the Fas/FasL signaling), a neoplastic origin, or an infection [8]. Different viruses have been linked with the disease, such as parvovirus B19, Epstein-Barr virus, and human herpes virus 6 [9].

The classical nodal disease is described as enlarged, painless bilateral cervical lymph nodes [6], night sweats, fever or weight loss, and leukocytosis. The mediastinal, axillary, inguinal, and para-aortic regions are other frequent sites for lymphadenopathies. Over 40% of patients may have an extranodal presentation, with symptoms depending on the compromised organ [9]; the most common areas are the skin, paranasal sinus, nasal cavity, eyelid, orbit, bone, salivary gland, and, less frequently, the central nervous system (CNS) [8,10].

CNS can be compromised in less than 5% of RDD cases and mainly affects adult men (mean age 39.5 years), with symptoms including headache, seizures, hemiparesis, cranial nerve deficits, and dysphasia developing over weeks to months [11]. Intracranial RDD affects the cerebral convexities (roughly 4.87%) [11], petroclival and parasagittal region, and cavernous sinus region. As in our case report, intracranial compromise may mimic a meningioma showing a solitary extra-axial, well-defined, dural-based lesion, with enhancing dural tails, isodense or hyperdense on CT [12], isointense to hypointense on T1, T2-weighted images, and fluid-attenuated inversion recovery MRI sequences [12-14]. RDD may show low relative cerebral blood volume in perfusion MRI, mild blooming on susceptibility-weighted imaging, or diffusion restriction on diffusion-weighted imaging [10]. FDG PET uptake may vary, with an avid FDG uptake from nodal and lacrimal disease and less FDG uptake for other sites [15]. In our patient, we found a mediastinal node with intensely avid FDG uptake that underwent robotic excision. The histopathology evaluation was consistent with RDD, suggesting that PET/CT should be considered for evaluating remaining activity lesions.

Histologically, there is lymphatic sinus dilatation due to histiocyte proliferation [5] and emperipolesis, in which histiocytes phagocytize lymphocytes, plasma cells, erythrocytes, or polymorphonuclear leukocytes [15]. The diagnosis can be firmly established using immunohistochemical markers. S100 protein is always positive, as well as CD68 and CD163 (histiocytic markers). Langerhans cell markers such as CD1a and CD207 are negative [7].

The clinical course is usually indolent, with spontaneous resolution of symptoms in many patients. However, RDD can present as a chronic disease with gradual progression, aggravation, or stabilization. Infrequently, RDD can take a more aggressive course compromising and destroying cartilaginous, bony, and soft-tissue structures [2,7]. RDD has no specific treatment as it resolves spontaneously. Surgery has been proposed for localized diseases, and radiation or chemotherapy for severe compromise with variable results [2].

## Conclusions

We report a case of RDD with brain and mediastinal involvement. Radiographic studies revealed a left parietal convexity extra-axial avidly enhancing mass, resembling a meningioma. Although history and imaging studies are essential for the initial approach, the histopathologic examination is crucial for identifying the disease. RDD is an uncommon condition but should be included in the differential diagnosis of brain lesions that consider a meningioma as a diagnosis, even more in patients from younger age groups and male sex. PET/CT is a strategic tool to rule out a synchronic compromise, as in this patient.
